# Patients’ Perceptions of Artificial Intelligence Acceptance, Challenges, and Use in Medical Care: Qualitative Study

**DOI:** 10.2196/70487

**Published:** 2025-05-15

**Authors:** Jana Gundlack, Carolin Thiel, Sarah Negash, Charlotte Buch, Timo Apfelbacher, Kathleen Denny, Jan Christoph, Rafael Mikolajczyk, Susanne Unverzagt, Thomas Frese

**Affiliations:** 1 Institute of General Practice and Family Medicine, Interdisciplinary Center of Health Sciences Medical Faculty of the Martin Luther University Halle-Wittenberg Halle (Saale) Germany; 2 Institute for Medical Epidemiology, Biometrics and Informatics, Interdisciplinary Center for Health Sciences Medical Faculty of the Martin Luther University Halle-Wittenberg Halle (Saale) Germany; 3 Institute for History and Ethics of Medicine, Interdisciplinary Center of Health Sciences Medical Faculty of the Martin Luther University Halle-Wittenberg Halle (Saale) Germany; 4 Friedrich-Alexander-Universität Erlangen-Nürnberg, Medical Informatics Erlangen Germany; 5 Junior Research Group (Bio-)medical Data Science Medical Faculty of the Martin Luther University Halle-Wittenberg Halle (Saale) Germany

**Keywords:** artificial intelligence, use, medical care, acceptance, patient, perceptions

## Abstract

**Background:**

Artificial intelligence (AI) is increasingly used in medical care, particularly in the areas of image recognition and processing. While its practical use in other areas is still limited, an understanding of patients’ needs is essential for the practical and sustainable implementation of AI, which could further acceptance of new innovations.

**Objective:**

The objective of this study was to explore patients’ perceptions toward acceptance, challenges of implementation, and potential applications of AI in medical care.

**Methods:**

The study used a qualitative research design. To capture a broad range of patient perspectives, we conducted semistructured focus groups (FGs). As a stimulus for the FGs and as an introduction to the topic, we presented a video defining AI and showing 3 potential AI applications in health care. Participants were recruited from different locations in the regions of Halle (Saale) and Erlangen, Germany; all but one group were from outpatient settings. We analyzed the data using a content analysis approach.

**Results:**

A total of 35 patients (13 female and 22 male; age: range 23-92, median 50 years) participated in 6 focus groups. They highlighted that AI acceptance in medical care could be improved through user-friendly applications, clear instructions, feedback mechanisms, and a patient-centered approach. Perceived key barriers included data protection concerns, lack of human oversight, and profit-driven motives. Perceived challenges and requirements for AI implementation involved compatibility, training of end users, environmental sustainability, and adherence to quality standards. Potential AI application areas identified were diagnostics, image and data processing, and administrative tasks, though participants stressed that AI should remain a support tool, not an autonomous system. Psychology was an area where its use was opposed due to the need for human interaction.

**Conclusions:**

Patients were generally open to the use of AI in medical care as a support tool rather than as an independent decision-making system. Acceptance and successful use of AI in medical care could be achieved if it is easy to use, adapted to individual characteristics of the users, and accessible to everyone, with the primary aim of enhancing patient well-being. AI in health care requires a regulatory framework, quality standards, and monitoring to ensure socially fair and environmentally sustainable development. However, the successful implementation of AI in medical practice depends on overcoming the mentioned challenges and addressing user needs.

## Introduction

In the absence of a common definition of artificial intelligence (AI), we used a broad approach in our study: AI involves using machines to simulate human reasoning and intelligent behavior, including thinking, learning, and reasoning with the aim of solving complex problems that can only be solved by human experts [1]. AI comprises machine learning (ML), deep learning, natural language processing, and computer vision [2].

The presence of AI has been steadily growing over the past few decades, particularly in developed countries, and has also expanded in health care in recent years [3,4]. In 2017, Esteva et al [5] published a study in which a neural network-based AI system outperformed dermatologists in the accuracy of diagnosing and classifying benign and malignant skin conditions. Several smartphone apps for patients promise accuracy in diagnosing skin lesions, but should rather be used for self-examination or in tele-dermatology [6]. By 2024, 692 AI/ML medical devices or algorithms were authorized and listed by the US FDA (Food and Drug Administration), mainly in radiology, followed by cardiology [7]. Such a database does not exist in Germany [8]. The German Federal Institute for Drugs and Medical Devices lists 65 digital health applications for patients that meet European standards for medical devices (CE-marked) and are reimbursed by public health insurance [9]. For most applications, however, it is not described whether they use AI or not. Approved medical devices in Germany using AI are, for example, applied for the detection of diabetic retinopathy [10] or for checking symptoms [11,12].

Although there are numerous approaches for AI-supported systems in medical care, their practical use seems limited [8]. This is partly due to a lack of adequate datasets and challenges in transferring developed systems into real-world applications [13]. If the discrepancy between theoretical development and practical implementation is not addressed, it may result in adverse outcomes and potential risks [13,14]. In this context, involving end users becomes crucial. Patient participation in health care and research has become increasingly important in recent years and has been highlighted as essential in numerous studies [15-19], as it not only enhances treatment outcomes [20] but also improves the quality of research [21]. Involving end users leads to a better understanding of their needs and better acceptance of new innovations. Otherwise, there is a risk of underuse, circumvention, or resistance to use [22,23]. The early engagement of patients and end users in AI research is essential for understanding their needs and identifying key points for education and practical applications [24], thereby enabling the development of practical and sustainable AI applications [25]. A qualitative methodology allows us to gather participants’ perceptions in an unbiased way, and especially to recognize the reasons for these perceptions to enhance understanding [26,27]. The exchange in focus groups and the stimulus given can trigger responses and allow participants to build on ideas that might not have come up in individual interviews [28]. Previous research has primarily used quantitative methods [29] or examined patients’ perceptions of specific medical applications or specializations [30,31]. This is also evident in Germany, where only a few studies exist [14,32-35]. Therefore, a qualitative methodology is needed, in addition to quantitative work in this field [36], to capture patients’ perspectives [37]. In addition, specific patient populations, including outpatients, older or chronically ill patients, and those from lower socioeconomic backgrounds, have been understudied [32,38,39]. To our knowledge, there has been no study that included these patient groups and used focus groups to explore general perceptions of AI in medical care.

There are several approaches for measuring the acceptance of technical innovations, including the Technology Acceptance Model (TAM) [40,41] and the Unified Theory of Acceptance and Use of Technology (UTAUT) [42], which have been predominantly used in previous studies, including in the health care sector [43-45]. In addition to the TAM, seven other models were combined in UTAUT by combining the following main factors that influence behavioral intention and usage behavior: performance expectancy, effort expectancy, social influences, and facilitating circumstances. The moderating factors of gender, age, voluntariness, and experience were also added [42].

The overarching aim of our research is to gain insight into a practical and reasonable implementation of AI in medical care by involving potential end users. Therefore, we aimed to examine patients’ attitudes and perceptions toward AI, regarding AI acceptance, challenges to AI implementation, and potential use in medical care, addressing the patient populations mentioned above.

## Methods

### Ethical Considerations

This qualitative study was conducted by a team of researchers from the universities of Halle-Wittenberg and Erlangen-Nürnberg, Germany, as part of the project “Perspectives on the Use and Acceptance of AI in Medical Care (PEAK)” and was approved by the medical faculty’s ethics committee of Martin Luther University Halle-Wittenberg (protocol 2021-229). After being informed of their rights, all participants provided written informed consent. We informed them that they could withdraw their consent at any time without providing a reason or facing any negative consequences. Participants were provided with snacks and drinks during the focus groups (FGs), though no financial compensation was offered. During transcription of the FGs, we used pseudonyms to maintain participants’ anonymity. We report our findings according to the Consolidated Criteria for Reporting Qualitative Research [46].

### Study Design, Participants, and Recruitment

We conducted semistructured FGs to capture patients’ perspectives on the acceptance, challenges, and use of AI in medical care. Participants were primarily selected through convenience sampling and further through purposive sampling and snowball sampling. We contacted participants directly and through study information leaflets and facility staff. We recruited our participants mainly in outpatient settings in and around Halle (Saale) and Erlangen, including university areas as well as family medicine and physiotherapy practices. We also included one FG of clinical patients in a psychiatric hospital in Erlangen to increase heterogeneity. Inclusion criteria for the study were defined as patients with first-hand experience of the German health care system, thus patients, who had at least one visit to either a general practitioner (primary care) or other outpatient medical specialists (secondary care) or a visit in a hospital (tertiary care) in their adult life (including current and previous visits). Patients younger than 18 years, lacking proficiency in German, or unable to consent were excluded.

### Examples and Focus Group Topic Guide

As a stimulus for the FGs, we (TA and JG) created a video defining AI and showing 3 potential health care applications: (1) diagnosis and symptom check (Ada app [Ada Health GmbH]) [11,12], (2) treatment (alternative medication plan) [47], and (3) process optimization in patient care (voice assistance) [48]. To reduce bias, we changed the presenting order of these examples in the FGs.

We (JG, SN, and CB) created a topic guide to generate open responses. We developed our guide using Krueger and Casey’s [49] approach to FG guides, Helfferich’s methods [50], the TAM [40,41], and the UTAUT [42].

Participants were asked the following questions:

1. What factors would make you more or less likely to accept an AI system in medical care?
2. What challenges do you see for a successful use of AI in medical care?
3. Where do you see potential applications for AI in medical care, and where don’t?

We pretested the topic guide and examples with colleagues and with the first FG of patients.

### Data Collection

Before the FGs, we collected participants’ sociodemographic data and health-related information. To assess technology affinity, the Perceived Technology Competence scale was used [51]. From June 2022 to March 2023, we conducted 6 FGs at university or medical practice locations with 5 to 8 participants each until thematic saturation was reached. FGs began with a video introduction to the topic, followed by discussions stimulated by our topic guide questions. The interviewer (JG) was a medical doctor, working as a researcher and doctoral candidate, and unknown to most participants. We informed participants about JG’s background and the aim of the study, and made sure that all participants had the opportunity to express their own opinions. We audio-recorded all FGs, which lasted between 86 and 134 minutes, and took field notes (Carsten Fluck [CF, research assistant in PEAK] and SN).

### Data Analysis

We systematically analyzed the textual material and categorized it using a content analysis approach [52]. To develop a category system, we (SN, CB, CF, and JG) independently coded one exemplary focus group, discussing and refining assigned text segments and categories until consensus was reached. JG and CF applied the category system to all FGs and debated changes after each FG until they reached a consensus. Based on the FG topic guide, we created main themes deductively, while we developed the subthemes inductively from the data. JG grouped coded segments together to identify key issues and to further structure the material. To ensure the quality criteria of validity and reliability of content analysis during the analysis [52], we compared our analysis tool with similar constructs from the literature (construct validity) and assumed that the category system and category definitions were appropriate (semantic validity) because the coded text passages were homogeneous in the respective categories. All researchers used MAXQDA 2022 (VERBI Software GmbH) for coding and analysis.

## Results

### Participants’ Characteristics and Experience With AI

Thirty-five patients participated, with a median age of 50 years, ranging from 23 to 92 years; most had chronic diseases. 13 identified as female and 22 as male. Participants’ socioeconomic status (SES) varied, with a trend toward higher SES. Participants frequently showed high and medium affinity for new technology (see Table 1).

**Table 1:** Characteristics of the participants (N=35).

Characteristic		Participants
Age (years), median (range)		50 (23-92)
**Gender, n (%)**		
	Female	13 (37)
	Male	22 (63)
**Highest education level, n (%)**		
	General qualification for university entrance (12-13 y)	19 (54)
	General certificate of secondary education (9-10 y)^a^	16 (46)
**Vocational qualification^b^, n (%)**		
	Completed vocational training	21 (60)
	In vocational training	3 (9)
	Advanced technical college certificate or university degree	15 (43)
	No vocational qualification	2 (6)
	Other vocational qualification	2 (6)
**Employment status, n (%)^c^**		
	Employed^d^	19 (54)
	Not employed	15 (43)
	Thereof pensioners	10 (29)
	Thereof students	2 (6)
**Chronic disease(s)^e^, n (%)**		
	Yes	20 (57)
	No	14 (40)
**Frequency of GP^f^ consultation^e^, n (%)**		
	Less than once every 3 months	15 (43)
	Once every 3 months	13 (37)
	Two to three times in 3 months	4 (11)
	Four times or more in 3 months	2 (6)
**Relationship to GP, n** **(%)**		
	Very good	18 (51)
	Rather good	13 (37)
	Neutral	1 (3)
	Rather poor	2 (6)
	Very poor	1 (3)
**Affinity for new technology^g^, n (%)**		
	Low	7 (20)
	Medium	12 (34)
	High	16 (46)

^a^Includes the German “Hauptschulabschluss.”

^b^Partially more than 1 vocational qualification exists.

^c^One participant in vocational training only.

^d^A total of 2 participants are simultaneously in vocational training and one participant is simultaneously in retirement.

^e^One participant did not respond.

^f^GP: general practitioner.

^g^Scale: Mean value of 3 items ≤2 low, >2 to <4 medium, and ≥4 high.

Most participants reported no experience with AI in medical care. A few stated they had had contact with or heard of AI in image recognition and processing (eg, computed tomography and magnetic resonance imaging), dental technology, pill reminder apps, care robots assisting with transport and entertainment, or surgical robots. However, surgical robots were more likely to be perceived as assistance systems that require human guidance.

The following sections present four main themes: (1) factors that promote the acceptance of AI systems in medical care, (2) factors that hinder the acceptance of AI systems in medical care, (3) patients’ perceived challenges and requirements for implementation, and (4) use of AI in medical care. Multimedia Appendix 1 shows the participants’ illustrative quotes for each subtheme.

### Theme 1: Factors Promoting Patients’ Acceptance of AI

Participants highlighted comprehensible instructions and explanations of the purpose of AI as beneficial, particularly among users with limited technical knowledge. Participants stated that AI should be easy to use and should improve personal life and patient care without imposing restrictions. Perceived enhancements were reduced costs and waiting times, and accelerated treatment. To guide system development, participants emphasized the importance of providing users with a permanent feedback opportunity to developers. Furthermore, participants indicated that AI should be based on a sufficiently large and representative database.

Ensuring transparency to understand the objectives of the development, data processing, and use of AI systems was identified as essential. Participants often lacked awareness or information about whether AI was running in the background, making it challenging to differentiate it from other technologies. Particularly with AI usage through several institutions, such as health insurance companies, transparency would allow patients to object to AI use.

Participants feared that high costs could disadvantage patients, who could not afford to use AI. Furthermore, they highlighted that the primary objective of AI should be to improve patient well-being, rather than focusing on commercial optimization. They suggested that financiers’ goals could influence AI development, leading to lobbying for more profitable options and potentially subpar medical care. Therefore, participants argued that nonprofit development and funding, for example, by an independent government body, would be crucial to ensure acceptance.

Well, I think the trick is, it has to be like, AI has to be geared towards patients. …And not commercially optimized. … The goal must be like: does it benefit the patient? If not, then it won't happen. That would actually be a good ethical approach, I think.

FG Participant 3

Participants felt the need for AI systems to be tested similarly to medical devices; testing with external validation and long-term use would be encouraging. In contrast, most participants did not want to be the first to have an AI system used on them.

A minor point raised was that the media and scientific institutes should provide objective information about AI systems, including details of testing, features, and focusing on positive patient and physician testimonials.

### Theme 2: Factors Hindering Patients’ Acceptance of AI

Participants considered human supervision and decision-making authority as a prerequisite for AI implementation, decreasing their acceptance if AI systems made decisions without human intervention. Human supervision was perceived as essential since the AI’s decision-making process is not comprehensible, and physicians could better assess whether the results are appropriate for individual patients.

In my opinion, a human should always make the decision. [FG Participant 5] Definitely, someone who checks what the AI has done.

FG Participant 2

Participants identified a possible lack of data protection and misuse of personal data as relevant barriers to the acceptance of AI systems. They emphasized the importance of privacy and data protection in medical applications from external access or trade, as misuse could also have negative effects on patients.

Participants stressed the importance of medical professionals’ attitudes toward AI systems, stating that if physicians were unconvinced or opposed to AI systems, participants' acceptance of these applications would decrease.

**Figure 1:**
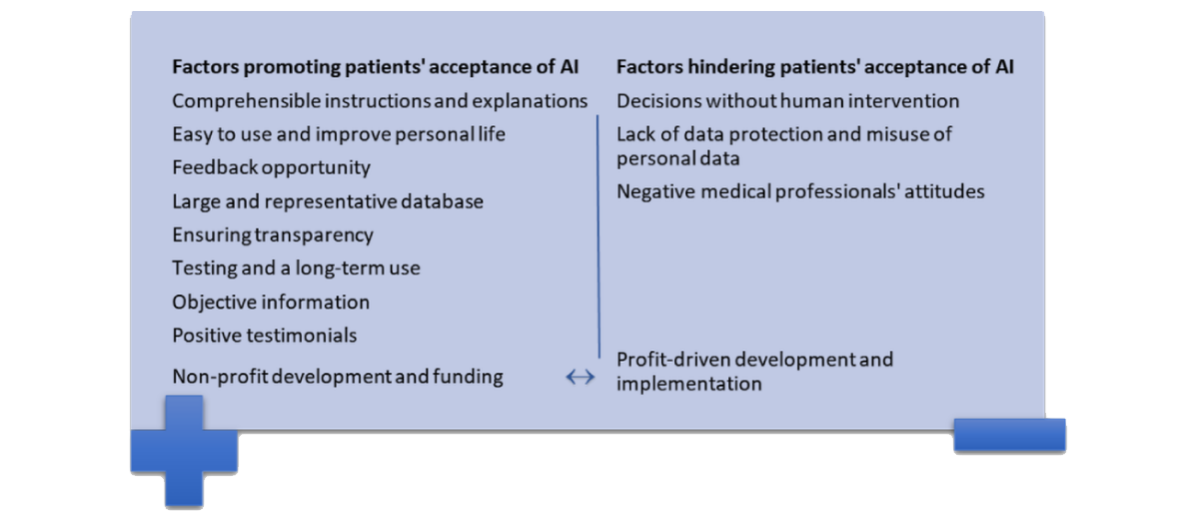
Factors promoting and hindering patients' acceptance of AI with respective subthemes. AI: artificial intelligence.

Figure 1 shows the subthemes of themes 1 and 2, and therefore, the factors that promote or hinder participants’ acceptance; Multimedia Appendix 1 shows the participants’ quotes.

### Theme 3: Challenges and Requirements for the Successful Use of AI in Medical Care

**Figure 2:**
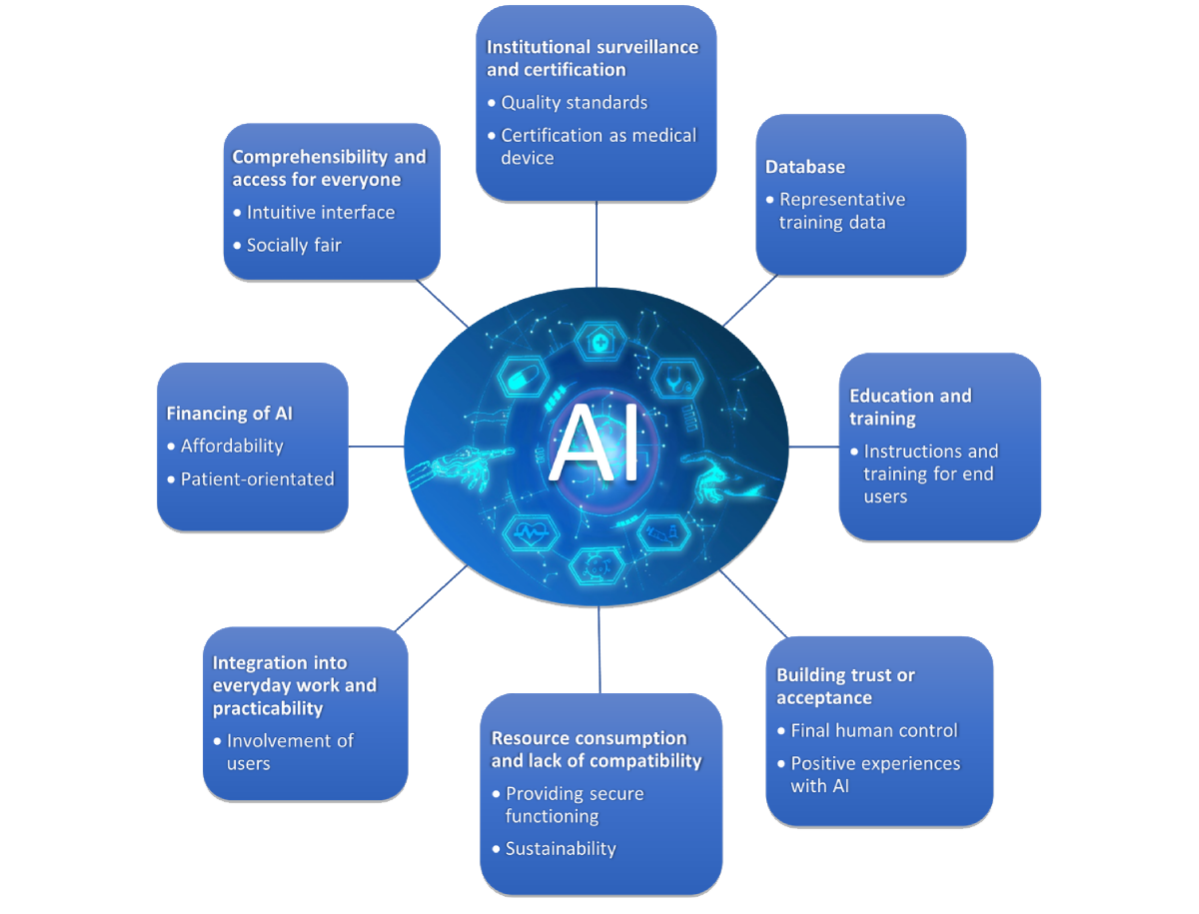
Subthemes of participants’ perceived requirements (headings) for artificial intelligence (AI) in medical care and resulting recommendations for practical development (bullet points).

The challenges mentioned were often also perceived as requirements for successful implementation, and are therefore presented in summary herewith (in addition, see Figure 2). We categorized 8 subthemes for this topic.

#### Resource Consumption and Lack of Compatibility

Participants pointed out that the widespread use of AI requires energy and human resources. Furthermore, AI requires substantial technical resources, which are currently not available ubiquitously, as the technical infrastructure, for example, the availability of software and hardware, varies between health care facilities, making widespread use and networking difficult. Participants supported regional and global networking for physicians and health care facilities to build databases and use AI systems, for example, to share information or coordinate treatment, but perceived the technical implementation as challenging. The lack of standardized software and incompatibility between existing systems were also regarded as challenges to the successful use of AI and networking. They emphasized the importance of compatibility between newly developed systems and those already in use.

So I think something like that could perhaps arise as a problem. With more providers and the more diversity, it might be more difficult to pick out a good system, and if they [doctors] want to exchange something with each other, that it's not all compatible.’

FG Participant 1

Therefore, participants considered balancing the provision of technology, and in particular, its practical functionality and maintenance, as a major challenge. Ensuring energy security and rapid technical service in the event of system failure was perceived as challenging but crucial, especially for medical applications on patients. Participants noted the need for human resources to develop and test AI, considering it difficult if additional staff were required to analyze and operate the AI, given the current staff shortage in the health sector. Participants also highlighted the importance of environmental sustainability in the context of digitalization and internet use, particularly in AI, which requires large servers for data collection and storage.

But you always have to consider sustainability. ...And if artificial intelligence is not sustainable, we'll have problems.

FG2 Participant 4

#### Comprehensibility and Access For Everyone

Participants stated that AI systems should have an intuitive interface, which is understandable to and usable for people without technical or medical expertise. Adaptable explanations, different languages, and support staff were suggested to ensure comprehensibility for all ages and levels of education. Participants also emphasized that AI systems should be accessible to all population groups.

I’d say accessibility above all. So that someone who is not tech-savvy, can simply still go there. And I have a menu or some kind of interface here that I can just use instinctively.

FG5 Participant 6

#### Education and Training

Participants emphasized the importance of user training and education for the successful application of AI, recommending short instructions and learning platforms for patients who use AI systems independently. They argued that physicians using AI would need training or qualifications, which they should arrange themselves, while others suggested that instructions for new AI systems should be available to physicians, considering integration into medical studies.

…[and for] the instruction to be so that it can be done, let’s say, in maybe ten minutes. So, the patient doesn't have to attend a course for a week to grasp the technology, because I think that would put most people off.

FG Participant 3

#### Financing of AI

According to participants, the development and use of AI could pose a noteworthy financial challenge. Participants questioned whether AI could be made available to everyone for free, and whether health insurance companies would subsidize AI applications.

And the other question is whether I could even pay for it. Whether I could pay for it at all.

FG2 Participant 4

#### Database

Participants identified the provision of sufficiently large amounts of recent and representative AI learning and working data as important. They expressed that a large number of training datasets would be necessary for adequate development and would therefore increase acceptance. In some cases, however, data provision was seen as difficult, for example, due to legal or time constraints from authorities or physicians.

The problem is learning data. I first need a huge amount of data to train it. Otherwise, I don’t get the precision I want.

FG3 Participant 4

#### Building Trust or Acceptance

Another challenge identified by participants was the need to build trust or acceptance of AI among patients and physicians, through time or positive experiences (see factors promoting acceptance). Participants stated that, as patients, they always had to trust the people treating them first, which would be no different with AI.

Acceptance from the users. Either doctors or patients. There has to be a certain level of acceptance. And it has to be built. Perhaps really through publications, information, education.

FG4 Participant 7

#### Integration Into Everyday Work and Practicability

A minor aspect mentioned was the challenges faced by health care professionals in integrating AI into their daily work, including the need for additional skills and time to explain and operate the technology. They suggested developing AI with a practical focus, involving users in the process, and gathering feedback to ensure easy integration into daily workflows.

Well, there has to be a benefit. Nobody is going to develop something that isn’t going to be useful or effective in practice in the end. ...So it has to be useful in practice somehow.

FG1 Participant 2

#### Institutional Surveillance and Certification

Participants preferred treating AI as a medical device, requiring approval before implementation. It was believed that current evaluations lacked oversight and independence, and suggested alternatives to assess AI systems based on medical benefits, applicability, and ethical considerations, rather than just economic factors. They advocated for transparent quality standards with defined goals and guidelines, and discussed the need for regulatory agencies or legal systems to set these guidelines. They also emphasized the need for oversight institutions monitoring AI operations to ensure compliance and consequences for noncompliance. Minor aspects mentioned were a legislation requiring humans rather than AI to make treatment decisions, as well as the role of government regulation and legal framework in promoting the fair use of AI without stifling development.

Well, that I have at least one institution that certifies or monitors the whole thing.

FG5 Participant 6

### Theme 4: Use of AI in Medical Care

#### Overview

The use of AI in medical care was a topic of debate for participants, with opinions ranging from its potential use everywhere, to being relevant only in clinical settings and not for individuals, to being entirely inconceivable. The dominant opinions were imagining AI as a support tool and information source, predominantly for physicians, and skepticism about AI making independent decisions, particularly in medicine, as this could involve health-related decisions and ethical considerations.

I’m a little tech-savvy. …But when it comes to medicine, my skepticism grows, to be honest. And there are a lot more ethical problems with medicine. Start small. Don’t immediately think of the doctor-AI doing everything.

FG4 Participant 7

#### Potential Future Areas of Application

Participants mentioned potential future areas of application throughout the treatment process (see the subthemes in Table 2). They considered AI to be beneficial in communicating with individuals who are unable (eg, by suggesting appropriate sentences) or afraid to speak with physicians (eg, due to anxiety or shame-inducing issues) and for adapting language levels or using different languages. Furthermore, AI could be used as a documentation support tool, for instance, during anamnesis or in the creation of drafts for physicians' letters.

**Table 2:** Subthemes and descriptions of potential and no potential areas of AI application mentioned by participants.

Themes and subthemes			Description of thinkable or unthinkable tasks provided by AI^a^
**Potential future areas of application**			
	Communication and documentation assistance	To assist in communicating medical history and structuring documentation.	
	Research, data collection, and networking	For a (long-term) structured collection and accelerating data transfer of patient data, findings, and diagnoses.	
	Diagnostics	As analysis tools for recording, processing, and monitoring patient values and alerting in case of disease development, as support in imaging procedures.	
	Therapy support and invasive interventions	As a support in medication planning, physiotherapy, rehabilitation exercises, and (remote) operations (mainly in surgery or orthopedics), for certain interventions with partly autonomous acting systems.	
	Care and everyday support for people in need of care	As robots to assist with manual tasks and care, and household activities, to record vital signs, for entertainment, including robotic animals.	
	Process management	To support processes in hospitals or practices.	
**No potential future areas of application**			
	Invasive interventions	Operating AI or robots that control themselves (especially in operations on vital organs or neurosurgery), or interventions directly on the human body (eg, taking of blood samples).	
	Care and direct patient contact	Activities requiring physical proximity or interpersonal relations, and entertainment or animal robots.	
	Empathic conversation (eg, in psychology)	Delivery and disclosure of serious illnesses or news; understanding, analyzing, assessing, and supporting the human psyche; psychotherapy; however, conceivable as support for conversations and medication regarding psychological illnesses.	
	Other specialties and therapy	Gynecology and urology; general medicine; sole therapeutic use, regardless of the specialty; in nonquantifiable examinations such as visual assessment or palpation.	

^a^AI: artificial intelligence.

Participants suggested that AI could be used in data collection and networking, building a database for physicians, and enabling early intervention through preventive monitoring of patient data. According to the participants, consolidating patient data and facilitating the exchange of expert knowledge could speed up treatment processes, identify correlations, and gain new insights. Furthermore, AI could support patient-physician coordination. For example, in research, AI could overcome language barriers to compile results for global studies.

Diagnostics was identified as a major field, as AI could accelerate the process through objective data analysis and collection, providing a basis for decision-making for physicians. Participants suggested including patient values in symptom checkers to improve diagnostic suggestions’ trustworthiness and reduce uncertainty. AI’s potential in image recognition, processing, and editing was expected to be leading in the near future (eg, in radiology, in neurology—evaluation of EEG data, in dermatology—screening of naevi), due to its already powerful capabilities in this area.

According to participants, AI could be used in therapy to help ensure that side effects, drug interactions, and current knowledge are taken into account. AI could be useful in providing rehabilitation and physiotherapy support by creating appropriate exercise plans with motivational elements, individual adaptation, and monitoring of exercises. Another possible area was operations, where a personal or verbal component seemed less important.

Participants discussed the use of AI in care and entertainment to address the shortage of skilled workers. Robots (featuring AI) could simplify work for carers and provide companionship for those in need of care. Beneficially, robotic animals would not cause allergies and would not have any physical needs. However, it was argued that AI alone should not be the answer; rather, it should be a change in approach to care to ensure enough carers.

Another area of application identified by participants was process management. They found AI useful in medication provision, operating theatre, and bed management, and patient triage. They identified great potential in administrative tasks, like optimizing ordering systems with faster appointment allocation, assisting patients with follow-up by providing necessary information and reminders, or physical assistance with luggage robots.

#### No Potential Future Areas of Application

Participants’ opinions differed regarding the potential use of AI in care and invasive interventions, ranging from possible (see above) to unthinkable. They expressed a lack of confidence in AI’s ability to perform operations reliably, as there would be no error tolerance. They could not imagine flexibility and short-term adaptation times (which would be required, for example, due to individual anatomy or the occurrence of errors) in the AI. Participants opposed AI in care, arguing that human interaction in care is crucial and should not be replaced by AI. They feared that vulnerable people in need of care (such as people with disabilities, children, and older adults) could be further excluded from society through the lack of human contact. There were comments about finding the idea of using care robots or animal robots sad, questioning how society will deal with people who need support in the future. In addition, operating these devices could also cost carers more time, potentially further reducing human contact.

Other areas that respondents felt were unsuitable for AI were tasks requiring empathic conversations, certain specialties, and sole AI use in therapy, regardless of specialty. Participants expressed distrust in AI’s ability to possess empathy and understanding of the human psyche (and its illnesses), which was mentioned as especially important in conversations. They also questioned whether AI could make appropriate therapy recommendations and provide support during difficult times. Participants highlighted that they would not want to be informed by AI about serious illnesses, or would be unsure how to deal with such a situation, because of the need for human contact in these settings. Participants opposed AI in gynecology or urology, either due to the sensitivity of health matters shared or for other unspecified reasons. AI in general medicine was also perceived as inappropriate, as patients often seek personal contact. A minor aspect was that research could not be imagined as a potential area for AI, as it requires human foresight. Subthemes with descriptions of thinkable and unthinkable tasks provided by AI are presented in Table 2, while participants’ quotes are presented in the Multimedia Appendix 1.

## Discussion

### Principal Findings

This study set out to examine patients’ perceptions of AI in medical care regarding acceptance, challenges, and use. According to participants, factors such as practicality, environmental sustainability, comprehensibility, accessibility for all, adherence to quality standards with proper monitoring, and a focus on patient well-being rather than profit should be considered in the development and implementation process. Though participants were not generally opposed to AI, there was some skepticism about its use, particularly in medicine. Participants could imagine AI as a support tool, but not as an autonomous system, indicating a desire for human control. Opinions diverged particularly on the use in care and operations. While diagnostics, including image recognition and processing, were seen as a dominant potential area of AI support, its use in areas where human interaction and conversation are essential was rejected.

### Comparison With Previous Work

#### Acceptance of AI

The UTAUT can help identify factors influencing user acceptance, especially among those hesitant to adopt new technologies, and can be applied in the development of technical innovations [42]. The results of our study regarding patients’ acceptance partly align with the main determinants of UTAUT (performance and effort expectancy, social influence, and facilitating conditions).

Participants expected AI to improve care or living conditions (performance expectancy). Important acceptance criteria included simple functionality and handling, and easy-to-understand explanations (effort expectancy), which were in line with previous studies [32,53]. Knowledge about AI can increase its acceptance [32,54], and both our participants and other stakeholders, including the European Commission, consider the transfer of knowledge and the training of medical staff and users to be challenging but indispensable prerequisites [55-59].

The participants valued recommendations from peers and physicians (social influences), stating negative attitudes from physicians would reduce their acceptance [60]. In line with the literature [61], participants’ acceptance would increase if AI was tested in studies and in practice, but no one wanted to be the first to test the system.

Factors promoting participants’ acceptance (facilitating conditions) were in line with previous studies and included data protection, patient- and nonprofit-oriented development and implementation [62,63] and a large and representative database for AI [63-65]. Previous studies have demonstrated that data protection and transparency in data use are essential for development, trust, and acceptance of AI [62,63], which is consistent with our findings. To prevent data leaks, robust security measures must be implemented, but this can hinder the acquisition of a sufficiently expansive and representative database [66]. In accordance with the literature, our participants viewed the attainment as a challenge, but an imperative requirement for AI to function adequately [63-65]. Furthermore, participants and current literature also discuss that AI systems should be trained on diverse data [67] to avoid inheriting existing inequalities from models or the training dataset [65]. Due to the sensitivity of health care data, participants emphasized its protection [68] and trustworthiness of entities receiving their data, which would be essential for data sharing [69]. Trustworthy AI should be in compliance with ethical and legal regulations as well as technical and social functioning throughout its lifecycle [70]. The US FDA, which approves medical AI devices, recommends cybersecurity, risk management, as well as postimplementation monitoring and evaluation, and calls for adaptive, science-based regulations that protect against risks without limiting benefits [71]. The European AI Act identifies AI in health care as a high-risk application, as it deals with personal data, and also emphasizes transparency, cybersecurity, and risk management throughout the AI lifecycle, and data governance to ensure representative and error-free training data [72]. Patients strongly opposed selling health data to private companies for AI research, despite some arguing it could be justified if the product benefits patients [73]. This distrust toward private health care companies was also evident in previous studies [74-76]. One of the most important findings was that participants desire independent AI development and funding aimed at patient benefit, with concerns being justified as AI may be used to increase profits [77]. The FDA also sees this risk, although it acknowledges that the relationship between financial optimization and improved health outcomes can be complex and result in financial disadvantages for provider organizations, insurance companies, or health systems. However, sponsors should be transparent and focus on health outcomes, and a comprehensive and regular approach across the health system is needed to counter the negative risks of financing and keep pace with the development of AI [71].

The UTAUT model provides a good orientation for measuring acceptance and intended use of AI. Due to the models’ criticized lack of complexity [43,78], UTAUT (and TAM) have been adapted and extended for use in the health care sector [79,80]. Nevertheless, it is only partially applicable to all health care issues and their stakeholders, including patients, physicians, and carers. Thus, as our results also show, sociocultural aspects or factors such as training or integration into everyday working life are important in health care applications [43,81] and need to be integrated into these models to reflect the complexity of digital applications in health care [45].

#### Challenges and Requirements

Participants emphasized that AI should be accessible and usable by all people, not exacerbating inequalities, as could be the case through biased training data, as mentioned above, or a lack of technical or financial possibilities [82].

In the context of resource use, the issue was mentioned that environmental sustainability should be considered when developing new AI systems. In a Swedish survey of health care managers, the climate aspect was mentioned in connection with the successful implementation of AI [58], and the European Commission identifies environmental sustainability throughout the lifecycle of AI as a requirement for trustworthy AI [56]. There are also efforts to assess the environmental compatibility of AI [83] and to identify the environmental impact of medical digitalization [84]. Participants considered environmental sustainability necessary, as a global digital infrastructure already consumes many resources and has a high carbon footprint. The participants' assumption is not unfounded: in 2019, 3.8 % of greenhouse gas emissions were attributed to the digital sector [85,86], and the trend is rising. AI has great potential to promote sustainable development and reduce the environmental footprint [4,87]. Yet it also has negative environmental impacts that require careful use and a balanced approach involving regulation and all stakeholders [88-90].

Comparison of our findings with existing literature confirms that patients prefer AI applications to be certified by external, independent institutions [62]. Participants and other health care stakeholders agreed that AI should meet quality standards, similar to medical devices [8,82]. As noted above, a regulatory framework was seen as crucial to the development and implementation of AI [58,65,91], guiding the development process without hindering progress and requiring oversight during application [65]. Assuming that AI could be fed with a global dataset in the future, then globally applicable regulations would be a logical consequence, although their implementation would undoubtedly prove challenging. Although there are recent developments, such as the European AI Act [72] or American regulatory approaches to AI in medical applications [71,91,92], trying to introduce compatible standards, there seems to be a lack of global guidelines.

#### AI Use in Medical Care

Despite participants expressing a general openness toward AI, there is considerable skepticism, even among tech-savvy participants, about AI-based decision-making and conversational guidance [73]. Particularly in the case of patients with chronic or terminal illnesses, the human factor appears to be of paramount importance [32,93]. Thus, some participants, who could imagine using AI for surgery, were against its independent use, but in favor of AI support [31]. In accordance with literature [94], opinions ranged from the independent use of AI for minor interventions to no use at all in surgical procedures.

As a key finding of our study and in line with previous studies, participants preferred AI as support systems with human supervision rather than autonomous systems [59,62,95]. As the lack of human involvement is a relevant barrier to acceptance of AI systems [61,95], potential applications should focus on support rather than independent functioning.

Our study indicates that most participants can imagine AI being used in diagnostics or data processing [96]. In line with literature [30,95], participants attributed a more objective diagnostic capability to AI and felt that AI could quicken the diagnostic process [30,60] and assist physicians, including in the identification of rare diseases [95].

AI-based systems already exist for outpatient and inpatient care [97]. Strikingly, there were substantial differences in participants' opinions regarding the use of AI in care. People in need of care may no longer be able to advocate for themselves, which increases the need for protection and raises ethical questions about the use of AI in care [97]. Furthermore, care is an intimate setting that involves both physical and emotional aspects, where maintaining communication and fostering a trusting relationship are of great importance [96]. Many participants felt that these aspects could not be provided by AI and therefore considered its use in care undesirable for activities beyond the manual relief of staff. In line with Deckert et al [96], they concluded that AI could only be integrated into care to a limited extent and could not replace nursing staff. Maintaining the interpersonal dimension would be a challenge [97] that skeptical participants felt AI could not meet. Other participants expressed the opposite view, arguing for the integration of AI in care, often stating that AI would be better than no contact at all. The diversity of opinions and concerns underscores the importance of a balanced approach to implementation in care that combines AI and humanity [97]. Furthermore, participants could not envisage AI in psychology, where conversational interactions are central. Nevertheless, some stated that certain patients might find it easier to confide in an AI than in medical staff concerning shameful issues or fears. Szalai [98] describes this aspect in the context of borderline therapy. Furthermore, conversational AI is advancing, as it is capable of engaging in a moderate conversation through language processing, using psychotherapeutic techniques [99]. Despite the existence of numerous potential applications for AI in mental health treatment, its actual use in clinical practice remains limited [97,100]. Most machine learning solutions for mental health are developed without involving end users and their individual needs, which can create barriers to using existing options [100]. Furthermore, there are concerns that human characteristics such as imperfection [101] or empathy [59] are necessary for psychological treatment, a perception shared by our participants. Exclusively technically generated treatment plans do not include the complete assessment by and emotional awareness of physicians [102]. This can critically reduce treatment success and discourage patients from continuing treatment [103]. AI has the potential to support mental health care, which seems particularly beneficial in light of the growing need for it [104]. However, in this specialist area, it seems particularly important to maintain basic ethical principles and physician involvement [104].

As we examined patients’ perceptions in two regions of Germany (south-west and central-east), and existing German studies in other health care settings have reported similar results [32-34], our findings are applicable to Germany as a whole. Patients’ perceptions of AI are also similar in comparison with other European countries [31,61] and industrialized nations [29,60].

### Strengths and Limitations

The qualitative design enabled an insight into patients’ perceptions and attitudes toward AI in medical care, and the focus groups contributed to a deeper discussion of the topic. Our questions, examples, and definition of AI merely provided a stimulus, allowing for open-ended responses and making this rather abstract topic more relatable. Yet these opinions should be taken into account in the development of new tools, as potential applications will be used on and by patients. A further strength of our study is the diversity of participants in terms of age, health history, technical affinity, and SES. In addition, the majority were outpatients, who have been underrepresented in previous studies. The wide range of participants made it possible to realistically reflect the perceptions of patients, helping to shape AI development in a practical way. In particular, the challenges and requirements highlighted will contribute to expanding the current state of knowledge and enable the sustainable development of new AI systems.

It is important to note that the sampling and recruitment process may introduce some selection bias into the results. As only patients who were interested in the topic and who tended to consider themselves tech-savvy participated, this may have strongly influenced their perceptions of AI and, therefore, the results. Although we tried to achieve a high level of diversity in terms of SES and affinity for technology, the majority of participants had a higher SES and a medium to high affinity for technology. The results may have limited applicability to populations with low socio-economic status and low affinity for technology, and these groups should continue to be addressed in future studies. As fewer people with a lower SES participated, it is possible that their opinions, especially their needs regarding AI systems in medical care, are underrepresented. Possibly, requirement priorities may differ for this group, such as secure funding, information pathways to reach this population, or the design of AI systems. Although participants stated that AI systems should be understandable and usable by people of all educational levels, this can only be ensured by explicitly asking all groups. In countries with substantial differences from German medical care or use of AI in health care, the applicability of our results is limited. In addition, it is possible that the topic guide questions and the examples provided influenced the patients' responses and the importance of topics. The medical background of the interviewer may have influenced the participants' responses, for example, by making them less open in their criticism of physicians or medical care, or by articulating more desirable topics such as support for medical staff. It is possible that due to the medical background, participants assumed a higher knowledge of AI in medical care and therefore tended to see themselves as inadequate participants, which may have led to more restraint. Most of the scenarios discussed were hypothetical, and the majority of participants were AI laypersons with no experience of AI in medicine and perhaps also with limited understanding of AI’s capabilities. Thus, the results should be interpreted accordingly. However, the perceptions of patients as laypersons are particularly interesting and relevant, as it is important to include end user views in the early development process.

### Future Work

The findings of this qualitative study, including the themes that patients identified as important, were used to develop a questionnaire for a subsequent quantitative study. It may therefore be possible to reach the aforementioned underrepresented patient groups, which we were able to address in this study, in greater numbers.

The perspectives of care recipients and patients from different ethnic backgrounds would be of particular interest for future research, as they have not been well studied. In addition, future studies need to examine the actual implementation of AI in health care settings and how the aforementioned requirements could be realized to ensure sustainable and fair development.

### Conclusions

Based on the results of our study, recommendations for developing patient-centered AI systems in medical care can be concluded. Recommendations for developers include practical development with user involvement and feedback, constant human control and final decision making, compatibility of newly developed systems with each other and with existing systems, sufficiently large and representative training data, ensuring transparency and data protection, comprehensible instructions and intuitive interface and usability, and adaptation to all age and education groups. Using AI as a supportive tool, rather than a replacement, and ensuring final human control was identified as crucial for implementing AI in medicine. Health care providers should learn about the capabilities of new systems before using them, so that they can evaluate the results of AI and explain the applications in use to patients. They should also maintain a human approach and be aware that their assessments of new AI systems will influence patients’ perceptions of AI. For their part, legislators should introduce clear quality standards and certifications to assess the trustworthiness of new AI systems as medical devices. The standards should include measures for socially fair and environmentally sustainable development and use, as well as education and training for users on system functions and practical use. At the same time, potential systems should be tested and verified for compliance with the standards before use, and compliance should be monitored. The most important guiding principle should always be patient welfare, not profit.

The successful implementation of AI systems in medical care faces many challenges, in part due to the prevailing caution and skepticism in this area. Nevertheless, as long as the development of AI systems is not primarily driven by profit, patients were generally open to their use and recognized their potential to support medical care. The extent to which they can be integrated into everyday medical practice will depend on whether the identified requirements and the needs of users will be taken seriously and whether the aforementioned challenges can be overcome.

## Appendix

Multimedia Appendix 1 Illustrative quotes organized by themes and subthemes.

## Abbreviations

AI: artificial intelligence
FDA: US Food and Drug Administration
FG: focus group
ML: machine learning
PEAK: Perspectives on the Use and Acceptance of AI in Medical Care
SES: socioeconomic status
TAM: Technology Acceptance Model
UTAUT: Unified Theory of Acceptance and Use of Technology

## Data Availability

The datasets generated and analyzed during this study are not publicly available as the participants’ anonymity and privacy may be at risk if all collected qualitative data is shared. The datasets are available from the corresponding author on reasonable request.
