# Supplementary material for: Patients’ Perceptions of Artificial Intelligence Acceptance, Challenges, and Use in Medical Care: Qualitative Study
Source: J Med Internet Res. 2025 May 15;27:e70487. doi: 10.2196/70487 (PMC12123243; doi:10.2196/70487)
Supplement: Multimedia Appendix 1 [file jmir_v27i1e70487_app1.docx]

Multimedia Appendix

Illustrative quotes organised by themes and subthemes.

| Themes and Subthemes | Illustrative Quotation |
| --- | --- |
|  |  |
| Factors promoting AI's acceptance in medical care | |
| *Comprehensible instruction*s/*explanations* | ‘But if it's new, I definitely need an instruction first and then how it works. And what the point of it is.’ (FG2-P.4) |
| *Easy to use and improve personal life* | ‘It must not hinder me." (FG-P.5) "It shouldn't be too complicated either.’ (FG-P.3) "It mustn't worsen the standard of living that I actually have. Good. It may improve it, but not worsen it. And then acceptance is certainly relatively high, in my opinion.’ (FG-P.5) |
| *Feedback to developers* | ‘...another very important point for me is, there must be permanent communication between the user and the developer. …so, people have to be able to give feedback on the knowledge they have gained from using the system.’ (FG1-P.5) |
| *Sufficiently large and representative data base* | ‘But that enough data was used, that this probability [AI works with] is somehow based on a large amount of data, that it [AI] is correct now.’ (FG3-P.5) |
| *Transparency* | ‘Yes, so there would be a bit of transparency again. Obviously, I don't have to understand exactly how it works. But you do need to be informed somehow: What can it do? Where are data records? Perhaps also, who developed this AI and why? (FG4-P.3) |
| *Non-profit development/funding* | ‘Well, I think the trick is, it has to be like, AI has to be geared towards patients. …And not commercially optimized. … The goal must be like: does it benefit the patient? If not, then it won't happen. That would actually be a good ethical approach, I think.’ (FG-P.3) |
| *Testing and long-term use* | ‘So, for one, it would be important to me that it is in use for a bit. So that it has been kind of validated over the years, for example, in a diagnostic tool. ...So that it's not just new on the market and then it's supposed to kind of tell me about my illness. I actually would like it to have been in use for a few years.’ (FG4-P.2) |
| *Objective information* | ‘And there has to be a conviction, and this is again a very important part, also of the world of media. And not somehow directed, but free. ... And in fact, objectively and not somehow led by emotions.’ (FG-P.5) |
| *Positive testimonials* | ‘If there are already a few evaluations or if the target rate is particularly high and the satisfaction is somehow already there, I think it would be easier to accept that.’ (FG3-P.5) |
| Factors hindering patients' acceptance of AI | |
| *Decisions without human intervention not desired* | ‘In my opinion, a human should always make the decision.’ (FG-P.5) ‘Definitely, someone who checks what the AI has done.’ (FG-P.2) |
| *Lack of data protection/Data misuse* | ‘And, where you can be sure that the data remains where it should be. That's also a really important point, of course.’ (FG-P.3) |
| *AI's development and implementation driven by profit* | ‘Well, it would be ok for me if a private company made money selling the system. But not if a company develops something and then continues to earn money from the certain diagnosis.’ (FG3-P.6) |
| *Medical professionals' attitudes* | ‘That’s a basic requirement for me. If the doctor says: “I don't really know”, then I don't think I would accept it. ... Well, I trust the doctor more than I would trust artificial intelligence. Perhaps that brings us back to personal relationships.’ (FG4-P.3) |
| Challenges and requirements for a successful use of AI in medical care | |
| Resource consumption and lack of compatibility | |
| *Technical, energy and partly*  *human resources* | ‘Well, someone has to develop and provide and implement the software first. Then it has to be used somehow. So, I think that's a really big challenge.’ (FG-P.1) |
| *Networking* | ‘It just means that with AI, all doctors should be connected with each other. So that the app knows it too. Or the database behind it. Basically, that would be ideal.’ (FG4-P.6) |
| *Lack of compatibility* | ‘So I think something like that could perhaps arise as a problem. With more providers and the more diversity, it might be more difficult to pick out a good system and if they [doctors] want to exchange something with each other, that it's not all compatible.’ (FG-P.1) |
| *Environmental sustainability* | ‘In any case, every technical invention is good and nice. But you always have to consider sustainability. ...And if artificial intelligence is not sustainable, we'll have problems.’ (FG2-P.4) |
| Comprehensibility and access for everyone | |
| *Intuitive interface* | ‘I’d say accessibility above all. So that someone who is not tech-savvy, can simply still go there. And I have a menu or some kind of interface here that I can just use instinctively.’ (FG5-P.6) |
| *Comprehensibility for all ages and*  *levels of education* | ‘And we should also look at what you suggested earlier in terms of the development and application of AI that different age groups should also be taken into account. Nowadays, children are ready. They can handle technical devices instantly. But older people, I don't know, 60, 70, 80, there are bound to be problems. And you also have to look at that: How can people handle it? Do I need something like big buttons? Touch screen. That sort of thing.’ (FG3-P.2) |
| Education and training | |
|  | ‘Of course, doctors also need to be trained. So, doctors can't teach themselves everything they need to know, but must also be instructed.’ (FG-P.4)  ‘[and for] the instruction to be so that it can be done, let's say, in maybe ten minutes. So, the patient doesn't have to attend a course for a week to grasp the technology, because I think that would put most people off.’ (FG-P.3) |
| Financing of AI | |
|  | ‘And the other question is whether I could even pay for it. Whether I could pay for it at all.’ (FG2-P.4) |
| Data base | |
|  | ‘The problem is learning data. I first need a huge amount of data to train it. Otherwise, I don't get the precision I want.’ (FG3-P.4) |
| Building Trust/ Acceptance | |
|  | ‘Acceptance from the users. Either doctors or patients. There has to be a certain level of acceptance. And it has to be built. Perhaps really through publications, information, education.’ (FG4-P.7) |
| Integration into everyday work and practicability | |
| *Integration into everyday work* | ‘The waiting room is full. Sometimes they don't even have the time. The doctor or GP must always have a little time to process this information and present it to the patient. The conversation. Time. That’s an important role for me.’ (FG3-P.3) |
| *Practicability* | ‘Well, there has to be a benefit. Nobody is going to develop something that isn’t going to be useful or effective in practice in the end. ...So it has to be useful in practice somehow.’ (FG1-P.2) |
| Institutional surveillance and certification | |
|  | ‘Well, that I have at least one institution that certifies or monitors the whole.’ (FG5-P.6) |
| Use of AI in medical care | |
| *Experiences with current*  *applications* | ‘So in the medical field in particular, AI is used in image processing to recognise a problem independently. For example, the retina, et cetera.’ (FG4-P.6) |
| *AI as a support tool*  *Scepticism about AI making*  *independent decisions, particularly*  *in medicine* | ‘You ‘d have to somehow say about AI: ‘Okay, I see it as a supporting instrument. Not as a replacement, but as a support.’ (FG5-P.5)  ‘I'm a little tech-savvy. I perhaps trust technology more than others. But regarding medicine, my scepticism grows, to be honest. Because there are a lot more ethical problems with medicine. Start small. Don't immediately think of the doctor-AI doing everything. Support.’ (FG4-P.7) |
| Potential future areas of application | |
| Communication  Documentation assistance | ‘Well, in parts I can really see it, especially when it comes to case history, admission and so on. It might be easier for some people to get things off their chest in front of a computer than in front of a person. Yes. regarding your own shame and everything.’ (FG-P.4)  ‘Discharge letter is also a key word. Sometimes the patient sits there for hours because: ‘The discharge letter isn't ready.’ I would be happy if AI was there to pre-draft the discharge letter. And then, when the doctor says: ‘Well, we've done it like this and that,’ then I’d say to myself as a patient: ‘Okay.’’ (FG3-P.2) we've done it this way and that way,’ then I say to myself as a patient: ‘Okay.’’ (FG3-P.2) |
| Research/Data collection  Networking | ‘Basically, as long as it's about any data collection and linking, there's nothing against it.’ (FG3-P.1) |
|  | ‘... if we now look at the medical centres. With a combination of different medical disciplines, where people, doctors from the different disciplines can access the data together and then draw their conclusions from it, where I say: ‘Okay, I have a network here between A diagnostics and B expert knowledge in order to make appropriate treatment suggestions.’.’ (FG1-P.5) |
| Diagnostics  Image processing/Recognition  and analysis | ‘I see a focus on diagnostics. ... the main problem ... is always the time between taking the sample and analysing it. And with certain diseases, this can sometimes be vital. And especially when I look at diagnostics, if I can quickly get results after testing, it's good for the doctor and for the patient.’ (FG1-P.5) |
|  | ‘Especially in the imaging field, EEG analysis, anywhere where technical data is collected somehow. Where you have some kind of technical device. And the data from it is analysed. That's where I see the task forces now.’ (FG4-P.5) |
| Therapy support  Invasive interventions | ‘I actually find this medication plan interesting. Because you don't always remember what treats what. And so you’d have an overview of everything, so to speak. And if someone thinks ahead. - you probably can't just use it without correcting it. But I ‘d say it's useful if it's already sorted.’ (FG3-P.5)  ‘I go to the rehab centre once a week. I have to do exercises and an exercise plan there. And I think you could do a lot there with AI techniques.’ (FG4-P.8) |
|  | ‘Because I could rather imagine being operated on by one [robot/AI] than having a robot talk to me about some essential things.’ (FG3-P.5) |
| Care | ‘Yes, quite simplified, to make everyday work and care easier.’ (FG5-P.1) |
| Process management  Administrative support | ‘Yes, it continues, if you look at the medication dilemma, with the procurement, delivery, and the order at the end. ...Artificial intelligence, you can probably improve a lot with that in terms of storage, stock, delivery, whatever, I guess.’ (FG1-P.2) |
|  | ‘Yes, for example, administrative things, for example, I can see that. The patient goes to the hospital. And you know how it is: they don't know their way around. They are then given one [robot] to take them there. That also carries his things. And delivers him to where he needs to go.’ (FG3-P.2) |
| No potential future areas of application | |
| Invasive interventions | ‘So in terms of operations, I can't imagine it with an AI. As there is no margin for error. 100 % or zero. I don't have the confidence in it.’ (FG4-P.8) ‘Yes, because a human being is not a standardised component.’ (FG4-P.7) |
| Care/Direct patient contact | ‘I also think something like physical contact and so on. Like, for example, caring for people or something like that. I don't think something like that should ever be given into the hands of AI or a robot-like being. ...Because that personal contact would simply be missing.’ (FG5-P.3) |
| Empathic conversation  Psychology/Psychiatry  Information about serious  diagnoses | ‘...especially with mental health, where I have to talk to the doctor so that the doctor can point this out to me and also gives me recommendations. I’d say: ‘I need someone here who can empathise with my problem. And who, if it's a bad constellation, will also help me out of this pit sensibly’ Machines won't be able to do that. I simply need the dialogue between doctor and patient.’ (FG1-P.5) |
|  | ‘If he tells me now: ‘So, palliative diagnosis.’ I don't want to hear that from a robot.’ (FG3-P.5) |
| Other specialties/Therapy | ‘But when it comes to therapy, I don't think that's possible solely via AI. In any area.’ (FG-P.4) |

Abbreviations: AI - artificial intelligence
